# Cryolipolysis and associated health outcomes, adverse events, and satisfaction: A systematic review and meta‐analysis

**DOI:** 10.1111/obr.13925

**Published:** 2025-04-11

**Authors:** Roshan Ravindran, Damiano Pizzol, Masoud Rahmati, Susanna Caminada, Dong Keon Yon, Jae Il Shin, Nicola Veronese, Pinar Soysal, Guillaume Fond, Laurent Boyer, José Francisco López‐Gil, Karel Kostev, Julia Gawronska, Lee Smith

**Affiliations:** ^1^ KLNIK, The Colony, Wilmslow Cheshire UK; ^2^ Health Unit, Eni San Donato Milanese Italy; ^3^ Health Unit, Eni Rome Italy; ^4^ School of Medicine ‐ La Timone Medical Campus, AP‐HM, Aix‐Marseille University, UR3279: Health Service Research and Quality of Life Center (CEReSS) Marseille France; ^5^ Department of Physical Education and Sport Sciences, Faculty of Literature and Humanities Vali‐E‐Asr University of Rafsanjan Vali‐E‐Asr University of Rafsanjan Iran; ^6^ CRSMP, Center for Mental Health and Psychiatry Research – PACA Marseille France; ^7^ Department of Pediatrics, Kyung Hee University Medical Center Kyung Hee University College of Medicine Seoul South Korea; ^8^ Department of Pediatrics Yonsei University College of Medicine Seoul South Korea; ^9^ Faculty of Medicine Saint Camillus International University of Health Sciences Rome Italy; ^10^ Department of Geriatric Medicine, Faculty of Medicine Bezmialem Vakif University Istanbul Turkey; ^11^ One Health Research Group, Universidad de Las Américas Quito Ecuador; ^12^ University Clinic Philipps‐University Marburg Marburg Deutschland Germany; ^13^ Centre for Health, Performance and Wellbeing Anglia Ruskin University Cambridge UK

**Keywords:** adiposity, adverse events, cryolipolysis, patient satisfaction

## Abstract

**Background:**

Cryolipolysis is a nonsurgical adiposity reduction treatment that is increasing in popularity globally. The aim of this paper was to carry out a systematic review with meta‐analysis on cryolipolysis and associated health outcomes, adverse events (AE) and patient satisfaction.

**Methods:**

Major databases were searched from inception until April 4, 2024. Meta‐analysis was performed using random‐effect models to calculate the pooled effects size and 95% confidence interval (CI) of each finding. The systematic review protocol was registered on PROSPERO, CRD‐42024548077.

**Results:**

A total of 30 studies were included, including 3158 participants. The result of meta‐analyses showed reduced body mass index (mean differences [MD] = −1.80, 95% confidence interval [CI] −2.98, −0.62, *p* = 0.0003), waist‐to‐hip ratio (MD = −0.09, 95% CI −0.16, −0.02, *p* = 0.001), mean abdominal circumference (cm) (MD = −3.56, 95% CI −4.98, −2.15, *p* = 0.000001), and mean suprailiac fat thickness (FT) (mm) (MD = −5.22, 95% CI −9.03, −1.42, *p* = 0.0007), 12 weeks after cryolipolysis as compared with baseline values. The satisfaction rate was 80.4% and the AE of cryolipolysis was 49.5% for numbness, 44.5% for erythema, 30.5% for edema, 28.8% for pain, 25.4% for sensitivity, 15.2% for tingling, and 2% for hyperpigmentation.

**Conclusion:**

In the present study, it was found that cryolipolysis was associated with a reduction in the number of adiposity parameters at 3 months follow‐up. A relatively high level of minor AEs was reported; however, patient satisfaction was high suggesting that the treatment is well tolerated.

## 1 INTRODUCTION

Non‐invasive procedures for fat reduction have become increasingly popular and requested as an aesthetic treatment over the last decade. Cryolipolysis (commonly known as CoolSculpting Allergan Aesthetics [Irvine, CA], an AbbVie Company [North Chicago, IL]) has become a cornerstone treatment for body sculpting treatments in aesthetic clinics as they can be done in office, with the oversight of a medical practitioner and little to no down time. Cryolipolysis is a nonsurgical technique that uses cold temperature to reduce fat deposits targeted at specific body regions.

In 1970, Epstein and Oren coined the term “popsicle panniculitis” after observing a red, firm nodule followed by temporary fat necrosis in the cheek of an infant who had been sucking on a popsicle.^1^ Originally described in infants, cold‐induced panniculitis has also been noted in adult patients.^2^ These findings suggest that fat‐rich tissues are more prone to cold injury than the surrounding water‐rich tissues. Inspired by these historical observations, Manstein et al. introduced a novel noninvasive fat‐reduction technique in 2007, known as cryolipolysis.^3^ This method involves applying a cooling device to the targeted area at a specific temperature for a set duration. This process targets fat cells while preserving the skin, nerves, blood vessels, and muscles.

Importantly, adipocytes are more susceptible to damage from cold temperatures than other cells. The cold temperature injures the adipocytes. The injury triggers an inflammatory response, which results in the apoptosis of the adipocyte cells. Macrophages then remove the dead fat cells and debris from the body and thus may reduce levels of adiposity.^4^ The beneficial aesthetic outcome of cryolipolysis is observed as a result of this selective disruption to adipocyte cells and their subsequent removal from the body. Owing to a potential reduction in adiposity, it is thus feasible that cryolipolysis has a positive impact on other health outcomes such as hypercholesterolemia.^5^ Other noninvasive device‐based body sculpting treatments, which differ in mode of action from cryolipolysis and are offered in aesthetic clinics include radio frequency (RF) and high‐intensity ultrasound (HIFU) or high‐intensity muscular stimulation (HIFEM). Additional minimally invasive injectable treatments such as deoxycholic acid (Aqualyx or Kybella) can also be considered.

These treatments are considered for patients who wish to avoid the downtime or cost of surgical liposuction, or the risks associated with such a procedure especially if the patient has concurrent comorbidities that may potentially render them unsuitable for surgical anesthetic intervention.

The US Food and Drug Administration has approved cryolipolysis to treat visible fat bulges in the submental and submandibular areas, thigh, abdomen, and flank, along with bra fat, back fat, underneath the buttocks, and upper arm. It is also FDA‐cleared to affect the appearance of lax tissue with submental area treatments.^6^ Cryolipolysis is increasingly sought as an alternative treatment to surgical fat‐removal procedures because it may produce high patient‐satisfaction levels without the same risks and recovery time.^7^, ^8^, ^9^, ^10^, ^11^, ^12^, ^13^, ^14^, ^15^ According to the Aesthetic Society's Aesthetic Plastic Surgery National Databank, nonsurgical fat‐reduction procedures totaled 140,314 in 2020 and were among the top 5 nonsurgical procedures performed in 2019.^16^, ^17^

There is also an emerging body of literature on potential adverse events (AE) in relation to cryolipolysis. Reported AEs include treatment site erythema, numbness/paresthesia, bruising, and oedema. With more serious complications including severe/persistent pain, dysesthesia, skin hyperpigmentation, motor neuropathy, and paradoxical adipose hyperplasia (PAH).^18^ These more serious complications question the utility of cryolipolysis. However, the true rate of such complications is not known.

Despite the potential health benefits of cryolipolysis and its emerging popularity among the general public, its influence on health outcomes, potential AEs, and patient satisfaction is not clearly known, as to date no attempt has been made to collate, synthesize, and evaluate the extent of the literature. Given this background, the aim of the present study was to carry out a systematic review and meta‐analysis on associations between cryolipolysis and studied health outcomes, AEs, and patient‐satisfaction levels.

## 2 MATERIALS AND METHODS

This systematic review and meta‐analysis adhered to the Preferred Reporting Items for Systematic Reviews and Meta‐Analyses (PRISMA)^19^ and Meta‐analysis Of Observational Studies in Epidemiology (MOOSE)^20^ statements and followed a structured protocol registered on International Prospective Register of Systematic Reviews (PROSPERO), CRD‐42024548077.

### 2.1 Data sources and literature search strategy

Two investigators (MR and DP) independently conducted a literature search using PubMed/MEDLINE, Scopus, Embase, and Web of Science from inception until April 4, 2024. Any inconsistencies were resolved by consensus with a third author (LS). In PubMed, the following search strategy was used: “CoolSculpting OR cryolipolysis OR lipocryolysis.”

Conference abstracts and reference lists of included articles were hand‐searched to identify any potential additional relevant work.

### 2.2 Study selection

Following the participants, intervention, controls, outcomes, and study design (PICOS) criteria, we included studies assessing the effects of cryolipolysis on health, AEs, and satisfaction in observational (case–control, cross‐sectional, cohort) studies.

Studies were excluded if the data were not analyzable; in vitro studies; or if they did not clearly report data regarding cryolipolysis effects on health, AEs, or satisfaction. No language restriction was a priori applied. Screening of the articles was independently performed by two reviewers (MR and DP), and discord was resolved through discussion with a third reviewer (LS).

### 2.3 Data extraction

For each eligible study, two independent investigators (MR and DP) extracted: name of the first author and year of publication, setting, sample size, mean age of the population, mean body mass index (BMI), mean abdominal circumference, skin folds, and fat thickness (FT) in different anatomic sites, biochemical blood parameters, patients satisfaction, and side effects such as pain, sensitivity disorders, edema, hyperpigmentation, erythema, numbness, and tingling. Any inconsistencies were resolved by consensus with a third author (LS).

### 2.4 Outcomes

The primary health outcomes considered were BMI, mean abdominal circumference, and mean skin fold suprailiac fat, from baseline to 3‐month follow‐up; further considered outcomes included patient‐satisfaction levels and AEs. All parameters were reported in the original papers as mean with standard deviations (SDs).

### 2.5 Assessment of study quality

Two independent authors (MR and LS) carried out the quality assessment of included studies' using the Newcastle‐Ottawa Scale (NOS).^21^ The NOS assigns a maximum of 9 points based on three quality parameters: selection, comparability, and outcome.^22^ Any inconsistencies were resolved by consensus with a third author (DP).

### 2.6 Data synthesis and statistical analysis

Outcomes were pooled and expressed as mean difference (MD) with corresponding 95% confidence intervals (CI) based on one‐stage approach and the random‐effects estimate using the DerSimonian‐Laird method. Moreover, the meta‐analysis prevalence of AE and rate of patient satisfaction was calculated using MedCalc 20.104 software (MedCalc®) with a 95% CI and random‐effect model. The degree of between‐study heterogeneity that could not be ascribed to sampling error was explored using Cochran's Q statistics and *I*‐squared (*I* ^2^; low: <25%, moderate: 25%–50%, moderate‐to‐substantial: 50%–75%, and substantial: >75%) to estimate heterogeneity. Further, the potential for publication bias was assessed using funnel plots with Egger's linear regression and Begg's rank tests, when the sufficient number of studies (*n* > 10) was available.^23^ Finally, to assess the robustness of summary estimates and to detect if any particular study accounted for a large proportion of heterogeneity, sensitivity analysis was performed by the one study removed method. All meta‐analyses in the current study were conducted using Review Manager (version 5.4; The Nordic Cochrane Centre, Copenhagen, Denmark) and MedCalc software version 20.104 (MedCalc software Ltd, Acacialaan 22 8400 Ostend‐Belgium). Finally, a two‐sided *p*‐value less than 0.05 was considered statistically significant.

## 3 RESULTS

### 3.1 Literature search

As shown in Figure 1,777 articles were initially screened, and 57 full texts were retrieved. Among them, 30 studies were finally included in the systemic review.

**FIGURE 1:**
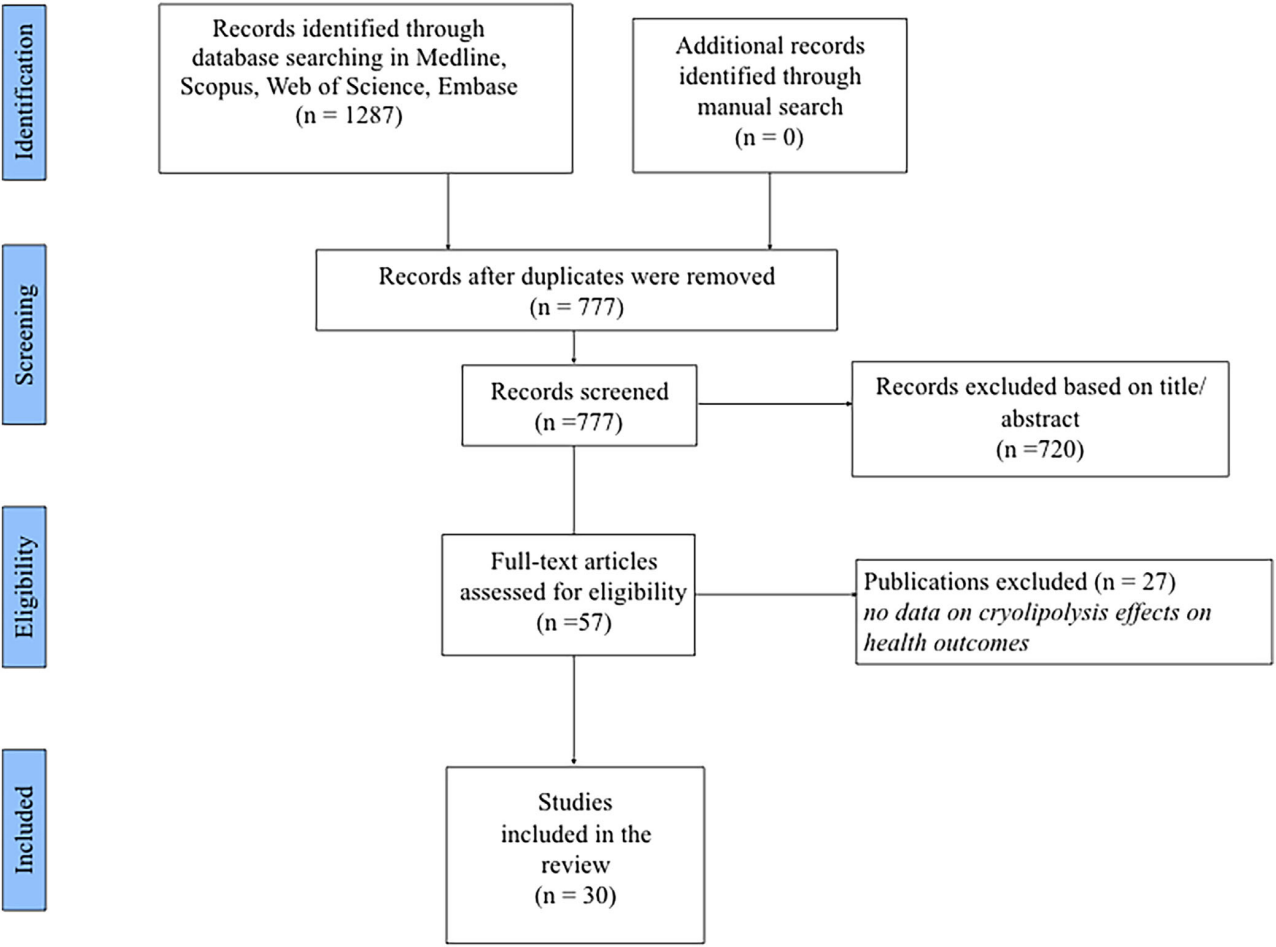
PRISMA flow diagram of study selection.

### 3.2 Descriptive findings and quality assessment

The majority of included studies were carried out in North America (*n* = 11) followed by Europe and Middle East (6 each), Asia (4), and South America (3). Overall, 3158 participants (range: 4–2114) were included having a mean age, for studies with available data, of 32.8 years (range: 14–51.4) (Table 1).

**TABLE 1:** General characteristics of included studies.

Study	Design	Country	Group (Gender: %F)	Age (year)	Number of treatments	Sites of treatment	Duration/temperature
Abdel‐Aal, 2020^24^	RCT	Egypt	30 (100)	45.7 ± 1.86	90	Hypogastrium	60 min/−5°C
Adjadj, 2017^11^	Cohort	France	48 (100)	38 ± 11	48	Saddlebags	55 min/−2°C
Altmann, 2022^25^	Cross‐sectional	Germany	91 (84)	45.5 ± NR	91	Various	NR
Azab, 2020^26^	Cohort	Egypt	46 (0)	37.2 ± 9.1	138	Suprapubic	30–60 min/0 to −5°C
Carruthers, 2017^27^	Cohort	Canada	60 (100)	45.7 ± 1.5	60	Arms	35 min/−11°C
Coiante, 2023^28^	Cohort	France	54 (100)	35 ± 1.3	108	Abdomen	55 min/−5°C
Eldesoky, 2015^29^	Case–control	Egypt	20 (70)	33.3 ± 5.33	80	Abdomen	30 min/NR
Falster, 2019^30^	RCT	Brazil	17 (100)	24.9 ± 5.04	17	Abdomen	50 min/−10°C
Faulhaber, 2019^31^	Cohort	Germany	4 (100)	33.8 ± 4.8	4	Abdomen or flank	NR
Faulhaber, 2019^31^	Cohort	Germany	10 (80)	40.4 ± 15.6	10	Abdomen or flank	NR
Garibyan, 2014^14^	RCT	USA	11 (55)	37.6 ± 8.4	11	Flank	60 min/NR
Hong, 2022^32^	Cohort	South Korea	15 (0)	33 ± 7.89	30	Breast	40 min/−7°C
Hwang, 2020^33^	RCT	South Korea	15 (30)	38.31 ± 10.84	15	Abdomen	60 min/−7°C
Jalian, 2022^34^	RCT	USA	16 (62.5)	43 ± NR	32	Submental	45 min/−11°C
Kandula, 2021^35^	Cohort	USA	12 (NR)	51.17 ± 6.5	12	Abdomen	Ice slurry 30% ice content
Keaney, 2015^36^	Cross‐sectional	USA	125 (78.4)	44.5 ± NR	554	Various	60 min/NR
Khedmatgozar, 2020^37^	Cohort	Iran	30 (100)	NR	30	Abdomen	60 min/−5°C
Kilmer, 2015^38^	Cohort	USA	60 (80)	NR	60	Submental	50 min/−10°C
Kilmer, 2016^39^	Cohort	USA	19 (NR)	46.7 ± NR	19	Flank	60 min/−10°C
Klein, 2009^40^	Cohort	USA	40 (80)	40.9 ± 10.5	40	Flanks	30 min/NR
Klein, 2017^41^	Cohort	USA	35 (77)	45.2 ± 2.2	35	Abdomen or flank	30 min/−10°C
Leal Silva, 2017^42^	Cohort	Mexico	15 (80)	46.2 ± NR	30	Submental	30–45 min/ −15–−12°C
Luze, 2022^43^	Cohort	Austria	29 (100)	39.5 ± 13.4	812	Abdomen	Belt, 1 h per day/18°C
Meyer, 2021^44^	RCT	Brazil	32 (100)	NR	64	Abdomen	75 min/−4°C
Mostafa, 2016^45^	RCT	Egypt	15 (60)	14 ± 1.9	120	Abdomen	60 min/NR
Mostafa, 2021^46^	RCT	Egypt	15 (60)	33.48 ± 3.91	45	Abdomen	60 min/NR
Nikolis, 2020^47^	Cross‐sectional	Canada	2114 (NR)	NR	8658	Various	NR
Ponga‐Manson, 2021^48^	Cohort	Spain	30 (100)	51.4 ± 3.4	150	Abdomen	60 min/−5°C
Savacini, 2018^49^	Cohort	Brazil	21 (85.7)	34 ± 9	NS	Abdomen and flanks	60 min/−8°C
Tan, 2021^50^	Cohort	Singapore	112 (74.1)	42.5 ± 9.86	NS	Abdomen and flanks	45 min/NR
Wanitphakdeedecha, 2015^51^	Cohort	Thailand	17 (100)	30.2 ± 5.8	40	Arms and thigs	60 min/NR

Abbreviations: NR, not reported; NS, not specified; RCT, randomized controlled trial.

The median quality of the studies was 5.8 (range: 4–8), indicating an overall good quality of the studies, according to the NOS (Table 2S).

**TABLE 2:** Results of the quantitative synthesis.

Factor	Total sample	MD/proportion	95% CI	*p*‐Value	Heterogeneity (*I* ^2^—*p*‐value)	Egger's test *p*‐value	Begg's test *p*‐value
BMI	256	−1.80	(−2.98, −0.62)	**0.003**	94%—0.00001	0.65	0.22
WHR	60	−0.09	(−0.16, −0.02)	**0.01**	96%—0.00001	0.78	0.52
Mean abdominal circumference (cm)	211	−3.56	(−4.98, −2.15)	**0.00001**	11%—0.35	0.76	0.22
Mean thigh circumference (cm)	102	−1.28	(−3.06, 0.49)	0.16	44%—0.18	NA	NA
Mean subcutaneous FT (mm)	131	−0.66	(−1.49, 0.16)	0.11	87%—0.004		
Mean thigh FT (mm)	83	−0.10	(−0.22, 0.02)	0.12	0%—0.84	NA	NA
Mean suprailiac FT (mm)	52	−5.22	(−9.03, −1.42)	**0.007**	89%—0.0001	0.55	0.12
Total cholesterol (mg/dL)	126	−7.75	(−25.79, 10.28)	0.40	90%—0.00001	0.58	0.31
Triglyceride (mg/dL)	126	−4.99	(−27.27, 17.28)	0.66	88%—0.0001	0.72	0.38
HDL (mg/dL)	126	5.07	(−10.12, 20.25)	0.51	98%—0.00001	0.64	0.14
LDL (mg/dL)	126	−5.85	(−21.24, 9.53)	0.46	91%—0.00001	0.78	0.65
ALT (mg/dL)	126	−2.51	(−10.27, 5.25)	0.53	93%—0.00001	0.52	0.54
AST (mg/dL)	126	−1.85	(−9.33, 5.53)	0.62	94%—0.00001	0.64	0.54
Abdominal sonography FT (cm)	79	−0.66	(−1.58, 0.26)	0.16	98%—0.00001	0.48	0.24
Satisfaction	479	80.4%	67.2–90.7	NA	90%—0.0001	0.8	0.18
Pain	324	28.8%	15.2–44.8	NA	86%—0.0001	0.17	0.09
Sensitivity	59	25.4%	13.9–98.2	NA	97%—0.0001	NA	NA
Hyperpigmentation	175	2%	0.006–7.53	NA	60%—0.08	0.14	0.12
Erythema	388	44.5%	22.2–68.1	NA	95%—0.0001	0.80	0.27
Edema	228	30.5%	14.8–49.1	NA	87%—0.0001	0.78	0.90
Numbness	217	49.5%	25.5–73.6	NA	92%—0.0001	0.50	1.00
Tingling	205	15.2%	4.5–30.5	NA	83%—0.0001	0.14	0.24

Abbreviations: ALT, alanine aminotransferase; AST, aspartate aminotransferase; BMI, body mass index; CI, coefficient interval; FT, fat thickness; HDL, high‐density lipoprotein, LDL, low‐density lipoprotein; MD, mean difference; NA, not applicable; WHR, waist‐to‐hip ratio.

### 3.3 Main outcomes

Overall pooled analyses showed reduced BMI (MD = −1.80, 95% CI ‐2.98, −0.62, *p* = 0.0003; Figure S1), WHR (MD = −0.09, 95% CI −0.16, −0.02, *p* = 0.001; Figure S2), mean abdominal circumference (cm) (MD = −3.56, 95% CI −4.98, −2.15, *p* = 0.000001; Figure S3), and mean suprailiac FT (mm) (MD = −5.22, 95% CI −9.03, −1.42, *p* = 0.0.007; Figure S4), 12 weeks after cryolipolysis as compared with baseline values (Table 2).

However, the beneficial effects of cryolipolysis 12 weeks after treatment did not reach to significancy for mean thigh circumference (cm) (MD = −1.28, 95% CI −3.06, 0.49, *p* = 0.16; Figure S5), mean subcutaneous FT (mm) (MD = −0.66, 95% CI −1.49, 0.16, *p* = 0.11; Figure S6), mean thigh FT (mm) (MD = −0.10, 95% CI −0.22, 0.02, *p* = 0.12; Figure S7), total cholesterol (mg/dL) (MD = −7.75, 95% CI −25.79, 10.28, *p* = 0.40; Figure S8), triglyceride (mg/dL) (MD = −4.99, 95% CI −27.27, 17.28, *p* = 0.66; Figure S9), HDL (mg/dL) (MD = 5.07, 95% CI −10.12, 20.25, *p* = 0.51; Figure S10), LDL (mg/dL) (MD = −5.85, 95% CI −21.24, 9.53, *p* = 0.46; Figure S11), ALT (mg/dL) (MD = −2.51, 95% CI −10.27, 5.25, *p* = 0.53; Figure S12), AST (mg/dL) (MD = −1.85, 95% CI −9.33, 5.53, *p* = 0.62; Figure S13), and abdominal sonography FT (cm) (MD = −66, 95% CI −1.58, 0.26, *p* = 0.16; Figure S14), as compared with baseline values.

The result of random‐effect meta‐analysis showed that the proportion of patient satisfaction was 80.4% (95% CI 67.2–90.7) (Figure S15). Moreover, the proportion of random‐effect meta‐analysis for AEs of cryolipolysis was 49.5% for numbness (95% CI 25.5–73.6) (Figure S16), 44.5% for erythema (95% CI 22.2–68.1) (Figure S17), 30.5% for edema (95% CI 14.8–49.1) (Figure S18), 28.8% for pain (95% CI 15.2–44.8) (Figure S19), 25.4% for sensitivity (95% CI 13.9–98.2) (Figure S20), 15.2% for tingling (95% CI 4.5–30.5) (Figure S21), and 2% for hyperpigmentation (95% CI 0.006–7.53) (Figure S22).

## 4 DISCUSSION

The present study is the first systematic review and meta‐analysis on the association between cryolipolysis and health outcomes, AEs, and patient satisfaction. A total of 30 studies were evaluated, including 3158 participants. The findings suggest that cryolipolysis is associated with significant reductions in BMI, WHR, mean abdominal circumference, and mean suprailiac fat, at 3 months of follow‐up. Such results indeed suggest that cryolipolysis is an effective nonsurgical treatment for reduction in some adiposity parameters. The study found a relatively high level of minor AEs with numbness, erythema, edema, and pain being the most frequent. Importantly, the present analysis found a high level of patient satisfaction in relation to the cryolipolysis procedure.

The mechanisms by which cryolipolysis reduce adiposity are well understood. When cryolipolysis is performed, it is thought that vacuum suction with regulated heat extraction impedes blood flow and induces crystallization of the targeted adipose tissue.^3^, ^52^ In turn, this cold ischemic injury may promote cellular injury in adipose tissue via cellular oedema, reduced Na‐K‐ATPase activity, reduced adenosine triphosphate, increased lactic acid levels, and mitochondrial free radical release.^53^ Moreover, the initial insult of crystallization and cold ischemic injury because of cryolipolysis is compounded by ischemia reperfusion injury, resulting in the release of reactive oxygen species, increased cytosolic calcium levels, and activation of apoptotic pathways.^53^ This crystallization and cold ischemic injury of adipocytes results in apoptosis and an inflammatory response, thus removal of adipocytes within the following weeks.^3^, ^54^, ^55^ Indeed, histological studies show that within 3 months, macrophages are mostly responsible for clearing the damaged cells and debris.^54^, ^56^ Moreover, both animal and human studies showed no abnormal lipid levels or liver function despite the induced adipocyte damage due to cryolipolysis.^57^

To date, there have been over 17+ million CoolSculpting® procedures (one piece of equipment used to carry out cryolipolysis) performed worldwide.^58^ Thus, the low‐risk safety profile of cryolipolysis is well established. The common minor and moderate localized AEs such as numbness, erythema, sensitivity, tingling, hyperpigmentation, edema, and pain^14^ have also been reported without representing particular concern and discomfort for patients with often spontaneous resolution.^38^, ^42^, ^51^ Rare moderate‐to‐severe complications described in the wider literature include PAH^59^ and cold burns.^60^ The development of PAH may be related to a combination of dynamics, including older models of cryolipolysis units and applicators, as well as individual characteristics that predispose certain patients.^47^ It is generally accepted that adherence to the correct protocols and procedures while using the newer CoolSculpting models per se reduce AEs. Importantly, reported patient satisfaction with cryolipolysis was high at 80.4%, suggesting that patients are overall satisfied with the outcomes of the procedure and suggesting the procedure is generally well tolerated.

Owing to the findings from this meta‐analysis cryolipolysis could be recommended for targeted adiposity reduction within the adult population. Importantly CoolSculpting states that the procedure is not a weight loss procedure; however, the meta‐analysis shows a significant reduction in BMI with cryolipolysis (MD = −1.80, *p* = 0.003). This challenges some of the current aesthetic community beliefs. While the present work shows that cryolipolysis as an independent treatment for adiposity reduction may be effective, a greater reduction in adiposity could be achieved with the combination of cryolipolysis with nutrition and targeted exercise intervention. However, to date there is a scarcity of research in this area. Moreover, future research is needed to understand the true efficacy of cryolipolysis across the lifespan, including older adults and whether adiposity reduction with cryolipolysis influences other health outcomes such as quality of life.

### 4.1 Strengths and limitations

This is the first study to systematically review and evaluate the literature on the relationship between cryolipolysis and health outcomes, AEs, and patient satisfaction and benefits from a relatively large number of studies and participant sample. The variety of search databases utilized, and extensive reference searches have reduced the risk of bias. However, findings from the present study must be interpreted in light of its limitations. Firstly, there is the potential influence of publication bias, with negative and null findings remaining in the “file drawer.” Secondly, only a few health outcomes in relation to cryolipolysis procedure have been studied to date, and its impact on other health outcomes such as quality of life have not been studied. Thirdly, data were recorded over a relatively short follow‐up period, 3 months; future studies should look to extend such follow‐up periods to 12 months and beyond to garner an understanding of the long‐term indications of this procedure.

## 5 CONCLUSIONS

In conclusion, in this systematic review with meta‐analysis consisting of 30 studies and 3158 participants, it was found that cryolipolysis was associated with a reduction in a number of adiposity parameters at 3 months follow‐up. Importantly a statistically significant reduction in BMI was observed, thus challenging the current paradigm thinking in aesthetics on this key health parameter. A relatively high level of minor AEs was reported in studies. However, patient satisfaction was high suggesting that the treatment is well tolerated and potentially effective for the treatment of localized fat reduction.

## CONFLICT OF INTEREST STATEMENT

Dr Ravindran provides CoolSculpting as a treatment option to patients in his clinics.

## Supporting information


**Table S1.** PRISMA 2020 Checklist
**Table S2**. Quality assessment and publication bias evaluation of included study using the Newcastle‐Ottawa Scale (NOS)
**Figure S1.** Meta‐analysis of the BMI 12 weeks after cryolipolysis as compared with baseline values.
**Figure S2.** Meta‐analysis of the WHR 12 weeks after cryolipolysis as compared with baseline values.
**Figure S3.** Meta‐analysis of the mean abdominal circumference (cm) 12 weeks after cryolipolysis as compared with baseline values.
**Figure S4.** Meta‐analysis of the mean suprailiac FT (mm) 12 weeks after cryolipolysis as compared with baseline values.
**Figure S5.** Meta‐analysis of the mean thigh circumference (cm) 12 weeks after cryolipolysis as compared with baseline values.
**Figure S6.** Meta‐analysis of the mean subcutaneous FT (mm) 12 weeks after cryolipolysis as compared with baseline values.
**Figure S7.** Meta‐analysis of the mean thigh FT (mm)12 weeks after cryolipolysis as compared with baseline values.
**Figure S8.** Meta‐analysis of the total cholesterol (mg/dl) 12 weeks after cryolipolysis as compared with baseline values.
**Figure S9.** Meta‐analysis of the triglyceride (mg/dl) 12 weeks after cryolipolysis as compared with baseline values.
**Figure S10.** Meta‐analysis of the HDL (mg/dl) 12 weeks after cryolipolysis as compared with baseline values.
**Figure S11.** Meta‐analysis of the LDL (mg/dl) 12 weeks after cryolipolysis as compared with baseline values.
**Figure S12.** Meta‐analysis of the ALT (mg/dl) 12 weeks after cryolipolysis as compared with baseline values.
**Figure S13.** Meta‐analysis of the ALT (mg/dl) 12 weeks after cryolipolysis as compared with baseline values.
**Figure S14.** Meta‐analysis of the abdominal sonography fat thickness (cm) 12 weeks after cryolipolysis as compared with baseline values.
**Figure S15.** Meta‐analysis results of the proportion of satisfaction.
**Figure S16.** Meta‐analysis results of the proportion of numbness.
**Figure S17.** Meta‐analysis results of the proportion of erythema.
**Figure S18.** Meta‐analysis results of the proportion of edema.
**Figure S19.** Meta‐analysis results of the proportion of pain.
**Figure S20.** Meta‐analysis results of the proportion of sensitivity.
**Figure S21.** Meta‐analysis results of the proportion of tingling.
**Figure S22.** Meta‐analysis results of the proportion of hyperpigmentation.
**Figure S23**. Funnel plot for publication bias: satisfaction
**Figure S24**. Funnel plot for publication bias: erythema.
