# Supplementary material for: Cryolipolysis and associated health outcomes, adverse events, and satisfaction: A systematic review and meta‐analysis
Source: Obes Rev. 2025 Apr 11;26(8):e13925. doi: 10.1111/obr.13925 (PMC12246886; doi:10.1111/obr.13925)

Cryolipolysis and associated health outcomes, adverse events and satisfaction: a systematic review and meta-analysis

Roshan Ravindran±[1], Damiano Pizzol*[2,3], Masoud Rahmati*[4], Susanna Caminada[3], Dong Keon Yon[5], Jae Il Shin[6], Nicola Veronese[7], Pinar Soysal[8], Guillaume Fond[4], Laurent Boyer[4], Jose Fancisco Lopez-Gil[9.10], Karel Kostev[11], Julia Gawronska,[12] Lee Smith±[12]

1. KLNIK, The Colony, Wilmslow, Cheshire, UK, SK9 4LY
2. Health Unit, Eni, San Donato Milanese, Italy
3. Caminada: 2 Health Unit, Eni, Rome, Italy
4. School of Medicine - La Timone Medical Campus, AP-HM, Aix-Marseille University, UR3279: Health Service Research and Quality of Life Center (CEReSS), Marseille, France
5. Department of Pediatrics, Seoul National University Hospital, Seoul National University College of Medicine, Seoul, 03080, South Korea
6. Department of Pediatrics, Yonsei University College of Medicine, Seoul, South Korea
7. Geriatric Unit, Department of Internal Medicine and Geriatrics, University of Palermo, Palermo, Italy
8. Department of Geriatric Medicine, Faculty of Medicine, Bezmialem Vakif University, Istanbul, Turkey
9. Department of Communication and Education, Universidad Loyola Andalucía, 41704 Seville, Spain
10. One Health Research Group, Universidad de Las Américas, Quito 170124, Ecuador
11. Epidemiology, IQVIA, 60549 Frankfurt, Germany
12. Centre for Health, Performance and Wellbeing, Anglia Ruskin University, UK

*Authors contributed equally

± Corresponding authors: KLINK, Altrincham Rd, Wilmslow SK9 4LY, UK, drrosh@klnik.co.uk; Anglia Ruskin University, East Rd, Cambridge CB1 1PT, UK, lee.smith@aru.ac.uk

Supplementary Materials

Contents

Table 1. PRISMA 2020 Checklist....3

Table 2. Quality assessment and publication bias evaluation of included study using the Newcastle-Ottawa Scale (NOS)....7

Figure 1S. Meta-analysis of the BMI 12 weeks after cryolipolysis as compared with baseline values. 8

Figure 2S. Meta-analysis of the WHR 12 weeks after cryolipolysis as compared with baseline values.....9

Figure 3S. Meta-analysis of the mean abdominal circumference (cm) 12 weeks after cryolipolysis as compared with baseline values. 10

Figure 4S. Meta-analysis of the mean suprailiac FT (mm) 12 weeks after cryolipolysis as compared with baseline values. 11

Figure 5S. Meta-analysis of the mean thigh circumference (cm) 12 weeks after cryolipolysis as compared with baseline values. 12

Figure 6S. Meta-analysis of the mean subcutaneous FT (mm) 12 weeks after cryolipolysis as compared with baseline values. 13

Figure 7S. Meta-analysis of the mean thigh FT (mm)12 weeks after cryolipolysis as compared with baseline values.....14

Figure 8S. Meta-analysis of the total cholesterol (mg/dl) 12 weeks after cryolipolysis as compared with baseline values. 15

Figure 9S. Meta-analysis of the triglyceride (mg/dl) 12 weeks after cryolipolysis as compared with baseline values.....16

Figure 10S. Meta-analysis of the HDL (mg/dl) 12 weeks after cryolipolysis as compared with baseline values. 17

Figure 11S. Meta-analysis of the LDL (mg/dl) 12 weeks after cryolipolysis as compared with baseline values. 18

Figure 12S. Meta-analysis of the ALT (mg/dl) 12 weeks after cryolipolysis as compared with baseline values. 19

Figure 13S. Meta-analysis of the ALT (mg/dl) 12 weeks after cryolipolysis as compared with baseline values. 20

Figure 14S. Meta-analysis of the abdominal sonography fat thickness (cm) 12 weeks after cryolipolysis as compared with baseline values. 21

Figure 15S. Meta-analysis results of the proportion of satisfaction. 22

Figure 16S. Meta-analysis results of the proportion of numbness.....23

Figure 17S. Meta-analysis results of the proportion of erythema.....24

Figure 18S. Meta-analysis results of the proportion of edema. 25

Figure 19S. Meta-analysis results of the proportion of pain....26

Figure 20S. Meta-analysis results of the proportion of sensitivity.....27

Figure 21S. Meta-analysis results of the proportion of tingling....28

Figure 22S. Meta-analysis results of the proportion of hyperpigmentation.....29

Figure 23S. Funnel plot for publication bias: satisfaction 30

Figure 24S. Funnel plot for publication bias: erythema.....31

Table 1. PRISMA 2020 Checklist

Section and Topic	Item #	Checklist item	Location where item is reported
**TITLE**			
Title	1	Identify the report as a systematic review.	1
**ABSTRACT**			
Abstract	2	See the PRISMA 2020 for Abstracts checklist.	3-4
**INTRODUCTION**			
Rationale	3	Describe the rationale for the review in the context of existing knowledge.	5
Objectives	4	Provide an explicit statement of the objective(s) or question(s) the review addresses.	5-6
**METHODS**			
Eligibility criteria	5	Specify the inclusion and exclusion criteria for the review and how studies were grouped for the syntheses.	7
Information sources	6	Specify all databases, registers, websites, organisations, reference lists and other sources searched or consulted to identify studies. Specify the date when each source was last searched or consulted.	7
Search strategy	7	Present the full search strategies for all databases, registers and websites, including any filters and limits used.	7
Selection process	8	Specify the methods used to decide whether a study met the inclusion criteria of the review, including how many reviewers screened each record and each report retrieved, whether they worked independently, and if applicable, details of automation tools used in the process.	7-8
Data collection process	9	Specify the methods used to collect data from reports, including how many reviewers collected data from each report, whether they worked independently, any processes for obtaining or confirming data from study investigators, and if applicable, details of automation tools used in the process.	7-8
Data items	10a	List and define all outcomes for which data were sought. Specify whether all results that were compatible with each outcome domain in each study were sought (e.g. for all measures, time points, analyses), and if not, the methods used to decide which results to collect.	8-9
	10b	List and define all other variables for which data were sought (e.g. participant and intervention characteristics, funding sources). Describe any assumptions made about any missing or unclear information.	8-9
Study risk of bias assessment	11	Specify the methods used to assess risk of bias in the included studies, including details of the tool(s) used, how many reviewers assessed each study and whether they worked independently, and if applicable, details of automation tools used in the process.	8-9
Effect measures	12	Specify for each outcome the effect measure(s) (e.g. risk ratio, mean difference) used in the synthesis or presentation of results.	9
Synthesis methods	13a	Describe the processes used to decide which studies were eligible for each synthesis (e.g. tabulating the study intervention characteristics and comparing against the planned groups for each synthesis (item #5)).	7-9
	13b	Describe any methods required to prepare the data for presentation or synthesis, such as handling of missing summary statistics, or data conversions.	N/A
	13c	Describe any methods used to tabulate or visually display results of individual studies and syntheses.	8-9
	13d	Describe any methods used to synthesize results and provide a rationale for the choice(s). If meta-analysis was performed, describe the model(s), method(s) to identify the presence and extent of statistical heterogeneity, and software package(s) used.	8-9
	13e	Describe any methods used to explore possible causes of heterogeneity among study results (e.g. subgroup analysis, meta-regression).	8

Section and Topic	Item #	Checklist item	Location where item is reported
	13f	Describe any sensitivity analyses conducted to assess robustness of the synthesized results.	8
Reporting bias assessment	14	Describe any methods used to assess risk of bias due to missing results in a synthesis (arising from reporting biases).	N/A
Certainty assessment	15	Describe any methods used to assess certainty (or confidence) in the body of evidence for an outcome.	N/A
**RESULTS**			
Study selection	16a	Describe the results of the search and selection process, from the number of records identified in the search to the number of studies included in the review, ideally using a flow diagram.	Figure 1
	16b	Cite studies that might appear to meet the inclusion criteria, but which were excluded, and explain why they were excluded.	Figure 1
Study characteristics	17	Cite each included study and present its characteristics.	Table 1
Risk of bias in studies	18	Present assessments of risk of bias for each included study.	Table S4
Results of individual studies	19	For all outcomes, present, for each study: (a) summary statistics for each group (where appropriate) and (b) an effect estimate and its precision (e.g. confidence/credible interval), ideally using structured tables or plots.	Figures 2-8,<br>Figures S1-S14
Results of syntheses	20a	For each synthesis, briefly summarise the characteristics and risk of bias among contributing studies.	16
	20b	Present results of all statistical syntheses conducted. If meta-analysis was done, present for each the summary estimate and its precision (e.g. confidence/credible interval) and measures of statistical heterogeneity. If comparing groups, describe the direction of the effect.	10-16
	20c	Present results of all investigations of possible causes of heterogeneity among study results.	10-16
	20d	Present results of all sensitivity analyses conducted to assess the robustness of the synthesized results.	11
Reporting biases	21	Present assessments of risk of bias due to missing results (arising from reporting biases) for each synthesis assessed.	Table S4
Certainty of evidence	22	Present assessments of certainty (or confidence) in the body of evidence for each outcome assessed.	N/A
**DISCUSSION**			
Discussion	23a	Provide a general interpretation of the results in the context of other evidence.	17-20
	23b	Discuss any limitations of the evidence included in the review.	20-21
	23c	Discuss any limitations of the review processes used.	20-21
	23d	Discuss implications of the results for practice, policy, and future research.	21
**OTHER INFORMATION**			

Section and Topic	Item #	Checklist item	Location where item is reported
Registration and protocol	24a	Provide registration information for the review, including register name and registration number, or state that the review was not registered.	N/A
	24b	Indicate where the review protocol can be accessed, or state that a protocol was not prepared.	6
	24c	Describe and explain any amendments to information provided at registration or in the protocol.	N/A
Support	25	Describe sources of financial or non-financial support for the review, and the role of the funders or sponsors in the review.	N/A
Competing interests	26	Declare any competing interests of review authors.	N/A
Availability of data, code and other materials	27	Report which of the following are publicly available and where they can be found: template data collection forms; data extracted from included studies; data used for all analyses; analytic code; any other materials used in the review.	Table 1

From: Page MJ, McKenzie JE, Bossuyt PM, Boutron I, Hoffmann TC, Mulrow CD, et al. The PRISMA 2020 statement: an updated guideline for reporting systematic reviews. BMJ 2021;372:n71. doi: 10.1136/bmj.n71
For more information, visit: http://www.prisma-statement.org/

Section and Topic	Item #	Checklist item	Reported (Yes/No)
**TITLE**			
Title	1	Identify the report as a systematic review and/or meta-analysis.	Yes
**BACKGROUND**			
Objectives	2	Provide an explicit statement of the main objective(s) or question(s) the review addresses.	Yes
**METHODS**			
Eligibility criteria	3	Specify the inclusion and exclusion criteria for the review.	Yes
Information sources	4	Specify the information sources (e.g. databases, registers) used to identify studies and the date when each was last searched.	Yes
Risk of bias	5	Specify the methods used to assess risk of bias in the included studies.	Yes
Synthesis of results	6	Specify the methods used to present and synthesise results.	Yes
**RESULTS**			
Included studies	7	Give the total number of included studies and participants and summarise relevant characteristics of studies.	Yes

Section and Topic	Item #	Checklist item	Reported (Yes/No)
Synthesis of results	8	Present results for main outcomes, preferably indicating the number of included studies and participants for each. If meta-analysis was done, report the summary estimate and confidence/credible interval. If comparing groups, indicate the direction of the effect (i.e. which group is favoured).	Yes
**DISCUSSION**			
Limitations of evidence	9	Provide a brief summary of the limitations of the evidence included in the review (e.g. study risk of bias, inconsistency and imprecision).	Yes
Interpretation	10	Provide a general interpretation of the results and important implications.	Yes
**OTHER**			
Funding	11	Specify the primary source of funding for the review.	No
Registration	12	Provide the register name and registration number.	No

From: Page MJ, McKenzie JE, Bossuyt PM, Boutron I, Hoffmann TC, Mulrow CD, et al. The PRISMA 2020 statement: an updated guideline for reporting systematic reviews. BMJ 2021;372:n71. doi: 10.1136/bmj.n71

For more information, visit: http://www.prisma-statement.org/

Table 2. Quality assessment and publication bias evaluation of included study using the New-castle-Ottawa Scale (NOS)

Study	Select 1	Select 2	Select 3	Select 4	Comparab **	Expos1	Expos2	Expos3	Total
Abdel-Aal, 2020	1	1	1	1	1	1	1	0	7
Adjadj, 2017	1	0	1	1	0	1	1	1	6
Altmann, 2022	1	0	0	1	0	1	0	1	4
Azab, 2020	1	0	1	1	0	1	1	1	6
Carruthers, 2017	1	0	1	1	0	1	0	1	5
Coiante, 2023	1	0	1	1	0	1	1	1	6
Eldesoky, 2015	1	1	0	1	1	1	1	0	6
Falster, 2019	1	1	0	1	2	1	1	0	7
Faulhaber, 2019	1	1	1	1	1	1	1	1	8
Garibyan, 2014	1	1	1	1	1	1	1	1	8
Hong, 2022	1	0	1	1	0	1	1	1	6
Hwang, 2020	1	1	1	1	1	1	1	0	7
Jalian, 2022	1	1	0	1	1	1	1	0	6
Kandula, 2021	0	0	1	1	0	1	0	1	4
Keaney, 2015	1	0	0	1	0	1	0	1	4
Khedmatgozar, 2020	1	1	1	1	1	1	1	1	8
Kilmer, 2015	1	0	1	1	0	1	0	1	5
Kilmer, 2016	1	0	1	1	0	1	0	1	5
Klein, 2009	1	0	1	1	0	1	0	1	5
Klein, 2017	1	0	1	1	0	1	1	1	6
Leal Silva, 2017	0	0	1	1	0	1	0	1	4
Luze, 2022	1	0	1	1	0	1	0	1	5
Meyer, 2021	1	1	0	0	1	1	1	0	5
Mostafa, 2016	1	1	1	1	1	1	1	1	8
Mostafa, 2021	1	1	1	1	1	1	1	0	7
Nikolis, 2020	1	0	0	1	0	0	1	1	4
Ponga-Manson, 2021	1	0	1	1	0	1	1	1	6
Savacini, 2018	1	0	1	1	0	1	0	1	5
Tan, 2021	1	0	1	1	0	1	1	1	6
Wanitphakdeedecha, 2015	1	0	1	1	0	1	1	1	6

Figure 1S. Meta-analysis of the BMI 12 weeks after cryolipolysis as compared with baseline values.

	3 Months			Baseline				Mean Difference
Study or Subgroup	Mean	SD	Total	Mean	SD	Total	Weight	IV, Random, 95% CI
Abdel-Aal, 2020	33.3	1	30	37.56	0.85	30	12.2%	-4.26 [-4.73, -3.79]
Adjadj, 2017	23.54	3.71	48	23.61	3.91	48	10.3%	-0.07 [-1.59, 1.45]
Coiante, 2023	23.32	3.41	54	23.62	2.1	54	11.3%	-0.30 [-1.37, 0.77]
Eldesoky, 2015	30.5	0.8	20	32.4	1	20	12.1%	-1.90 [-2.46, -1.34]
Hwang, 2020	25.47	2.53	15	25.64	2.76	15	9.4%	-0.17 [-2.06, 1.72]
Khedmatgozar, 2020	28.05	3.06	30	29.55	3.08	30	10.2%	-1.50 [-3.05, 0.05]
Luze, 2022	26.9	1.7	29	27.1	1.8	29	11.6%	-0.20 [-1.10, 0.70]
Mostafa, 2016	25.3	0.8	15	28.5	1.5	15	11.6%	-3.20 [-4.06, -2.34]
Mostafa, 2021	29.3	1.3	15	33.2	1.5	15	11.4%	-3.90 [-4.90, -2.90]
**Total (95% CI)**			**256**			**256**	**100.0%**	**-1.80 [-2.98, -0.62]**

Heterogeneity: Tau² = 2.91; Chi² = 126.14, df = 8 (P < 0.00001); I² = 94%
Test for overall effect: Z = 2.99 (P = 0.003)

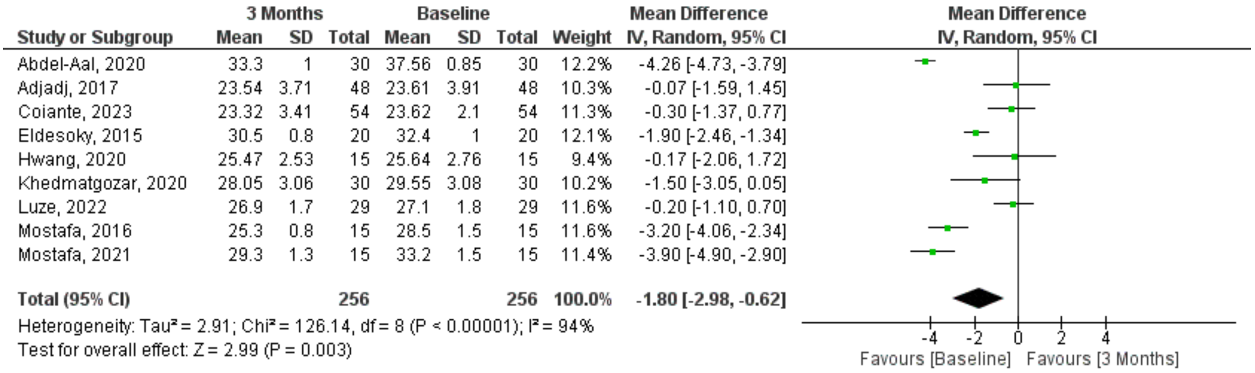

Figure 2S. Meta-analysis of the WHR 12 weeks after cryolipolysis as compared with baseline values.

	3 Months			Baseline				Mean Difference
**Study or Subgroup**	**Mean**	**SD**	**Total**	**Mean**	**SD**	**Total**	**Weight**	**IV, Random, 95% CI**
Abdel-Aal, 2020	0.8	0.04	30	0.93	0.03	30	34.0%	-0.13 [-0.15, -0.11]
Hwang, 2020	0.91	0.05	15	0.91	0.04	15	32.4%	0.00 [-0.03, 0.03]
Mostafa, 2016	0.81	0.0074	15	0.95	0.043	15	33.6%	-0.14 [-0.16, -0.12]
**Total (95% CI)**			**60**			**60**	**100.0%**	**-0.09 [-0.16, -0.02]**

Heterogeneity: Tau² = 0.00; Chi² = 55.93, df = 2 (P < 0.00001); I² = 96%
Test for overall effect: Z = 2.51 (P = 0.01)

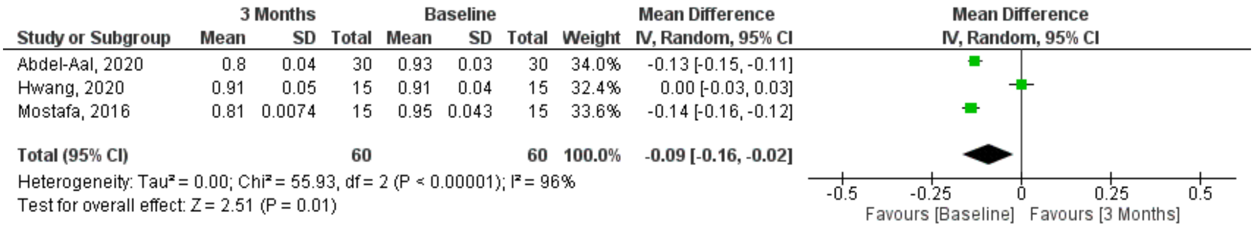

Figure 3S. Meta-analysis of the mean abdominal circumference (cm) 12 weeks after cryolipolysis as compared with baseline values.

	3 Months			Baseline				Mean Difference
**Study or Subgroup**	**Mean**	**SD**	**Total**	**Mean**	**SD**	**Total**	**Weight**	**IV, Fixed, 95% CI**
Coiante, 2023	93.61	5.65	54	95.6	9.5	54	23.1%	-1.99 [-4.94, 0.96]
Eldesoky, 2015	97.55	4.9	20	104.3	5.51	20	19.2%	-6.75 [-9.98, -3.52]
Falster, 2019	88.5	5.83	17	89.56	5.1	17	14.8%	-1.06 [-4.74, 2.62]
Faulhaber, 2019	86	8.2	4	88.5	8.2	4	1.6%	-2.50 [-13.86, 8.86]
Faulhaber, 2019 (2)	93.1	10.1	10	97.5	11.5	10	2.2%	-4.40 [-13.89, 5.09]
Hwang, 2020	88.52	10.66	15	91.18	7.87	15	4.5%	-2.66 [-9.37, 4.05]
Khedmatgozar, 2020	89.92	8.04	30	95.97	7.61	30	12.8%	-6.05 [-10.01, -2.09]
Luze, 2022	88.1	7.6	29	90	8	29	12.5%	-1.90 [-5.92, 2.12]
Meyer, 2021	80.3	9.4	32	84.4	9.6	32	9.3%	-4.10 [-8.76, 0.56]
**Total (95% CI)**			**211**			**211**	**100.0%**	**-3.56 [-4.98, -2.15]**

Heterogeneity: Chi² = 8.96, df = 8 (P = 0.35); I² = 11%
Test for overall effect: Z = 4.93 (P < 0.00001)

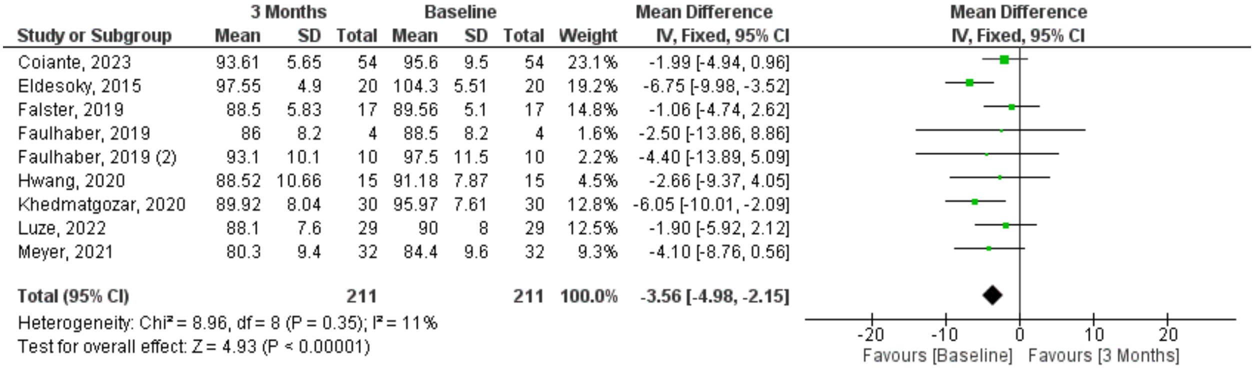

Figure 4S. Meta-analysis of the mean suprailiac FT (mm) 12 weeks after cryolipolysis as compared with baseline values.

	3 Months			Baseline				Mean Difference
**Study or Subgroup**	**Mean**	**SD**	**Total**	**Mean**	**SD**	**Total**	**Weight**	**IV, Random, 95% CI**
Eldesoky, 2015	25.14	2.79	20	30.44	2.9	20	35.2%	-5.30 [-7.06, -3.54]
Falster, 2019	35.5	5.1	17	36.6	4	17	30.5%	-1.10 [-4.18, 1.98]
Mostafa, 2016	11.2	2.1	15	20	3.4	15	34.4%	-8.80 [-10.82, -6.78]
**Total (95% CI)**			**52**			**52**	**100.0%**	**-5.22 [-9.03, -1.42]**

Heterogeneity: Tau² = 9.90; Chi² = 17.67, df = 2 (P = 0.0001); I² = 89%
Test for overall effect: Z = 2.69 (P = 0.007)

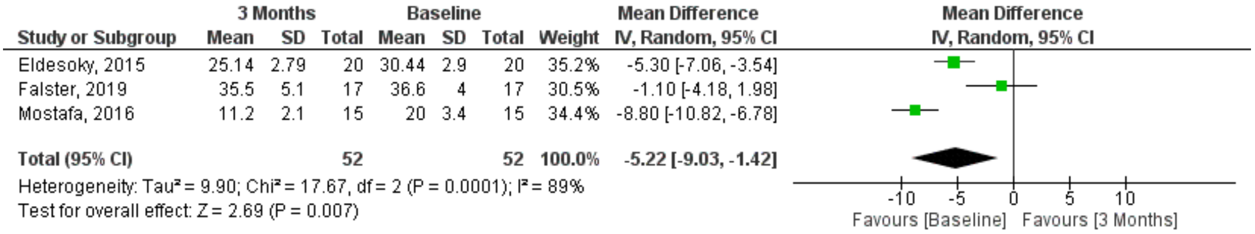

Figure 5S. Meta-analysis of the mean thigh circumference (cm) 12 weeks after cryolipolysis as compared with baseline values.

	3 Months			Baseline				Mean Difference
**Study or Subgroup**	**Mean**	**SD**	**Total**	**Mean**	**SD**	**Total**	**Weight**	**IV, Fixed, 95% CI**
Altmann, 2022	99.75	5.75	48	102.56	8.31	48	38.5%	-2.81 [-5.67, 0.05]
Coiante, 2023	71.52	6.13	54	71.85	5.85	54	61.5%	-0.33 [-2.59, 1.93]
**Total (95% CI)**			**102**			**102**	**100.0%**	**-1.28 [-3.06, 0.49]**

Heterogeneity: Chi² = 1.78, df = 1 (P = 0.18); I² = 44%
Test for overall effect: Z = 1.42 (P = 0.16)

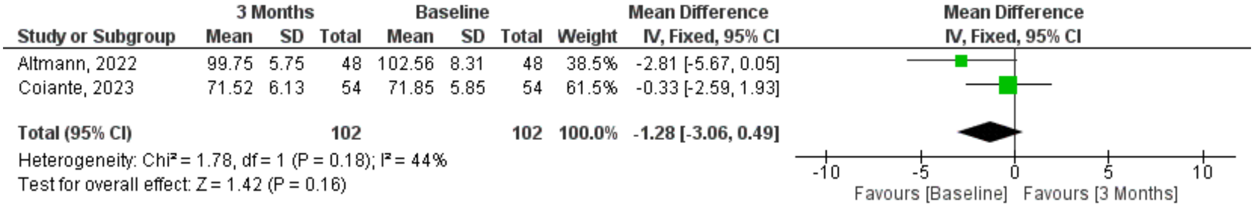

Figure 6S. Meta-analysis of the mean subcutaneous FT (mm) 12 weeks after cryolipolysis as compared with baseline values.

Study or Subgroup	3 Months Mean	3 Months SD	3 Months Total	Baseline Mean	Baseline SD	Baseline Total	Weight	Mean Difference IV, Random, 95% CI
Adjadj, 2017	2.45	1.25	48	3.4	1.51	48	33.9%	-0.95 [-1.50, -0.40]
Coiante, 2023	2.45	2.35	54	3.7	2.51	54	26.8%	-1.25 [-2.17, -0.33]
Luze, 2022	1.25	0.35	29	1.27	0.34	29	39.3%	-0.02 [-0.20, 0.16]
**Total (95% CI)**			**131**			**131**	**100.0%**	**-0.66 [-1.49, 0.16]**

Heterogeneity: Tau² = 0.44; Chi² = 15.57, df = 2 (P = 0.0004); I² = 87%
Test for overall effect: Z = 1.58 (P = 0.11)

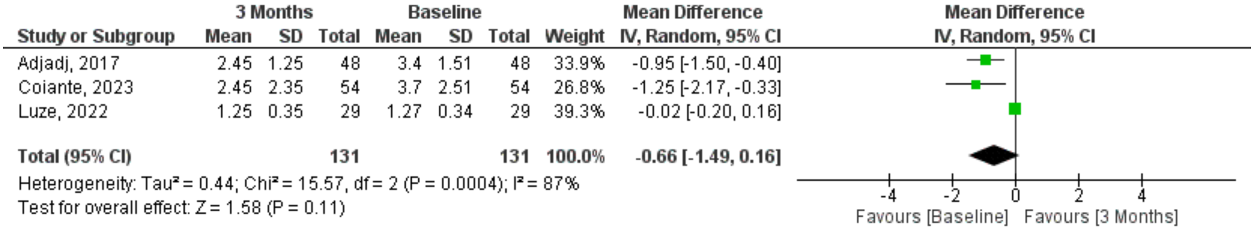

Figure 7S. Meta-analysis of the mean thigh FT (mm)12 weeks after cryolipolysis as compared with baseline values.

	3 Months			Baseline				Mean Difference
**Study or Subgroup**	**Mean**	**SD**	**Total**	**Mean**	**SD**	**Total**	**Weight**	**IV, Fixed, 95% CI**
Coiante, 2023	2.43	2.35	54	2.44	2.25	54	2.0%	-0.01 [-0.88, 0.86]
Luze, 2022	0.9	0.23	29	1	0.25	29	98.0%	-0.10 [-0.22, 0.02]
**Total (95% CI)**			**83**			**83**	**100.0%**	**-0.10 [-0.22, 0.02]**

Heterogeneity: Chi² = 0.04, df = 1 (P = 0.84); I² = 0%
Test for overall effect: Z = 1.57 (P = 0.12)

Mean Difference
IV, Fixed, 95% CI

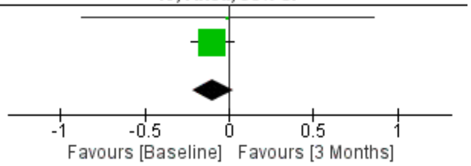

Figure 8S. Meta-analysis of the total cholesterol (mg/dl) 12 weeks after cryolipolysis as compared with baseline values.

Study or Subgroup	3 Months Mean	3 Months SD	3 Months Total	Baseline Mean	Baseline SD	Baseline Total	Weight	Mean Difference IV, Random, 95% CI
Abdel-Aal, 2020	219.63	8.26	30	244.1	6.34	30	29.0%	-24.47 [-28.20, -20.74]
Klein, 2009	177.1	26.5	40	173.3	23.1	40	26.5%	3.80 [-7.09, 14.69]
Klein, 2017	189.2	39.8	35	186.5	39.1	35	22.4%	2.70 [-15.78, 21.18]
Savacini, 2018	166.1	25.54	21	176.4	36.35	21	22.1%	-10.30 [-29.30, 8.70]
**Total (95% CI)**			**126**			**126**	**100.0%**	**-7.75 [-25.79, 10.28]**

Heterogeneity: Tau² = 288.57; Chi² = 30.62, df = 3 (P < 0.00001); I² = 90%
Test for overall effect: Z = 0.84 (P = 0.40)

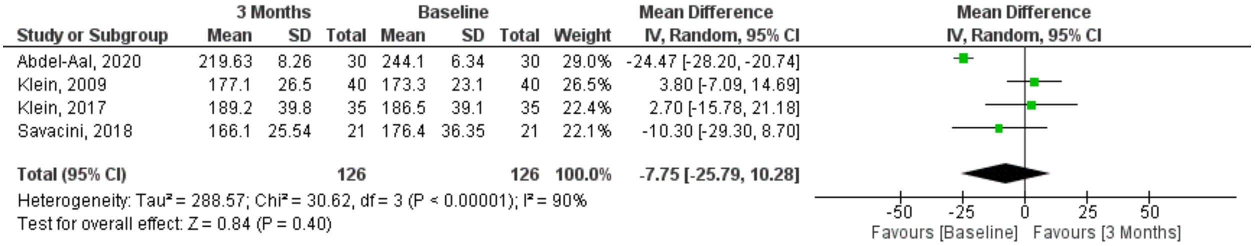

Figure 9S. Meta-analysis of the triglyceride (mg/dl) 12 weeks after cryolipolysis as compared with baseline values.

	3 Months			Baseline				Mean Difference
Study or Subgroup	Mean	SD	Total	Mean	SD	Total	Weight	IV, Random, 95% CI
Abdel-Aal, 2020	163.57	12.34	30	187.87	11.67	30	30.1%	-24.30 [-30.38, -18.22]
Klein, 2009	93.2	40	40	82.1	30.3	40	26.8%	11.10 [-4.45, 26.65]
Klein, 2017	83.4	38.6	35	77	32.5	35	26.2%	6.40 [-10.32, 23.12]
Savacini, 2018	89.18	57.42	21	103	63.16	21	16.9%	-13.82 [-50.33, 22.69]
**Total (95% CI)**			**126**			**126**	**100.0%**	**-4.99 [-27.27, 17.28]**

Heterogeneity: Tau² = 419.32; Chi² = 25.64, df = 3 (P < 0.0001); I² = 88%
Test for overall effect: Z = 0.44 (P = 0.66)

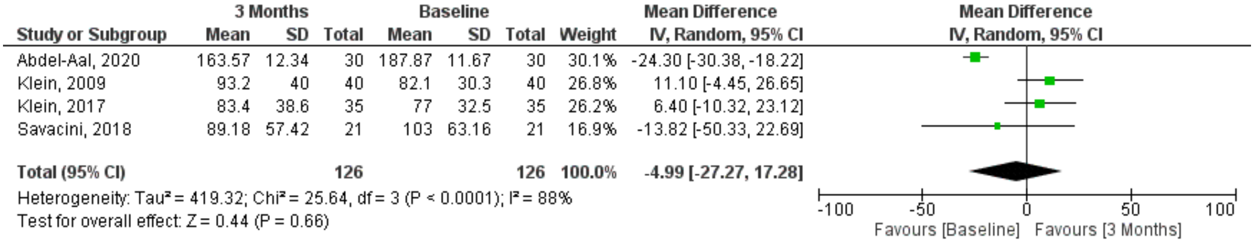

Figure 10S. Meta-analysis of the HDL (mg/dl) 12 weeks after cryolipolysis as compared with baseline values.

Study or Subgroup	3 Months Mean	3 Months SD	3 Months Total	Baseline Mean	Baseline SD	Baseline Total	Weight	Mean Difference IV, Random, 95% CI
Abdel-Aal, 2020	56.1	4.18	30	35.33	5.11	30	26.3%	20.77 [18.41, 23.13]
Klein, 2009	66.7	11.6	40	67	11.4	40	25.7%	-0.30 [-5.34, 4.74]
Klein, 2017	73.8	31.7	35	71	26.8	35	21.8%	2.80 [-10.95, 16.55]
Savacini, 2018	47.09	7.22	21	50.65	1.01	21	26.2%	-3.56 [-6.68, -0.44]
**Total (95% CI)**			**126**			**126**	**100.0%**	**5.07 [-10.12, 20.25]**

Heterogeneity: Tau² = 226.67; Chi² = 169.49, df = 3 (P < 0.00001); I² = 98%
Test for overall effect: Z = 0.65 (P = 0.51)

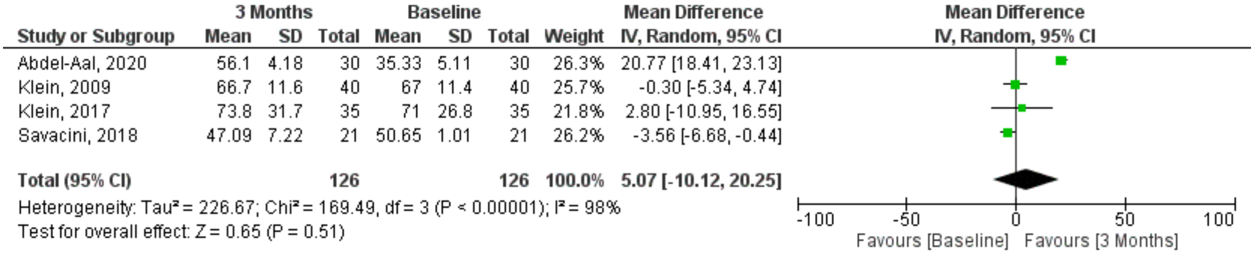

Figure 11S. Meta-analysis of the LDL (mg/dl) 12 weeks after cryolipolysis as compared with baseline values.

	3 Months			Baseline				Mean Difference
**Study or Subgroup**	**Mean**	**SD**	**Total**	**Mean**	**SD**	**Total**	**Weight**	**IV, Random, 95% CI**
Abdel-Aal, 2020	156.2	9.69	30	178.37	10.1	30	27.8%	-22.17 [-27.18, -17.16]
Klein, 2009	91.8	23.7	40	89.8	18.9	40	25.9%	2.00 [-7.39, 11.39]
Klein, 2017	102.5	28.7	35	100	13.1	35	25.3%	2.50 [-7.95, 12.95]
Savacini, 2018	101.2	26.45	21	105.2	30.49	21	21.0%	-4.00 [-21.26, 13.26]
**Total (95% CI)**			**126**			**126**	**100.0%**	**-5.85 [-21.24, 9.53]**

Heterogeneity: $Tau^2 = 215.11$; $Chi^2 = 32.13$, df = 3 ($P < 0.00001$); $I^2 = 91\%$

Test for overall effect: Z = 0.75 (P = 0.46)

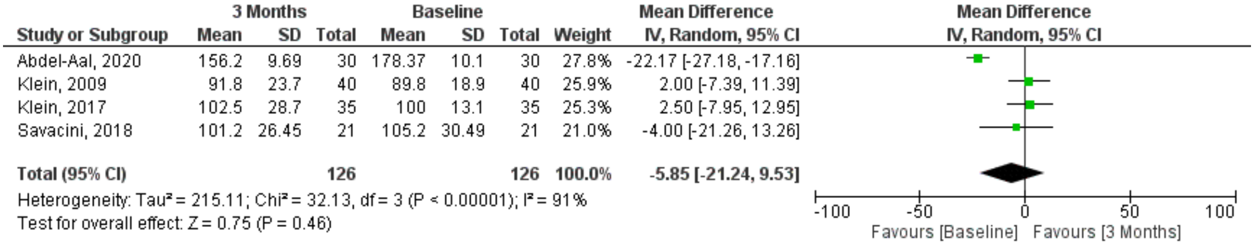

Figure 12S. Meta-analysis of the ALT (mg/dl) 12 weeks after cryolipolysis as compared with baseline values.

	3 Months			Baseline				Mean Difference
Study or Subgroup	Mean	SD	Total	Mean	SD	Total	Weight	IV, Random, 95% CI
Abdel-Aal, 2020	62.67	4.69	30	75.8	8.06	30	26.2%	-13.13 [-16.47, -9.79]
Klein, 2009	15.9	6.4	40	17.1	6.6	40	26.5%	-1.20 [-4.05, 1.65]
Klein, 2017	19.4	15.3	35	17.7	7.5	35	24.0%	1.70 [-3.95, 7.35]
Savacini, 2018	21.27	8.71	21	17.67	11.96	21	23.3%	3.60 [-2.73, 9.93]
**Total (95% CI)**			**126**			**126**	**100.0%**	**-2.51 [-10.27, 5.25]**

Heterogeneity: $Tau^2 = 56.98$; $Chi^2 = 41.76$, $df = 3$ ($P < 0.00001$); $I^2 = 93\%$
Test for overall effect: $Z = 0.63$ ($P = 0.53$)

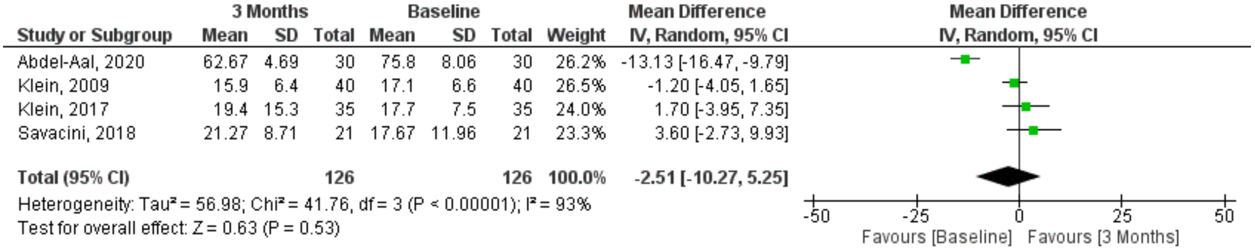

Figure 13S. Meta-analysis of the ALT (mg/dl) 12 weeks after cryolipolysis as compared with baseline values.

	3 Months			Baseline				Mean Difference
Study or Subgroup	Mean	SD	Total	Mean	SD	Total	Weight	IV, Random, 95% CI
Abdel-Aal, 2020	46.57	4.75	30	59.6	8.65	30	25.4%	-13.03 [-16.56, -9.50]
Klein, 2009	19.6	6.6	40	19.2	5.5	40	26.0%	0.40 [-2.26, 3.06]
Klein, 2017	23	16.3	35	19.7	5.2	35	23.2%	3.30 [-2.37, 8.97]
Savacini, 2018	21.27	5.9	21	18.96	5.83	21	25.4%	2.31 [-1.24, 5.86]
**Total (95% CI)**			**126**			**126**	**100.0%**	**-1.85 [-9.23, 5.53]**

Heterogeneity: $Tau^2 = 52.63$; $Chi^2 = 49.61$, df = 3 (P < 0.00001); $I^2 = 94\%$
Test for overall effect: Z = 0.49 (P = 0.62)

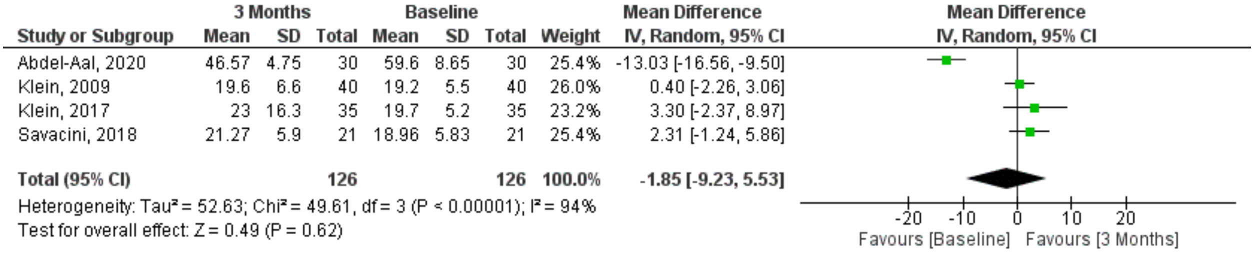

Figure 14S. Meta-analysis of the abdominal sonography fat thickness (cm) 12 weeks after cryolipolysis as compared with baseline values.

	3 Months			Baseline				Mean Difference
**Study or Subgroup**	**Mean**	**SD**	**Total**	**Mean**	**SD**	**Total**	**Weight**	**IV, Random, 95% CI**
Abdel-Aal, 2020	2.49	0.09	30	3.84	0.04	30	34.2%	-1.35 [-1.39, -1.31]
Falster, 2019	3.33	0.44	17	3.33	0.4	17	33.2%	0.00 [-0.28, 0.28]
Meyer, 2021	1.9	0.6	32	2.5	0.8	32	32.6%	-0.60 [-0.95, -0.25]
**Total (95% CI)**			**79**			**79**	**100.0%**	**-0.66 [-1.58, 0.26]**

Heterogeneity: Tau² = 0.64; Chi² = 103.13, df = 2 (P < 0.00001); I² = 98%
Test for overall effect: Z = 1.40 (P = 0.16)

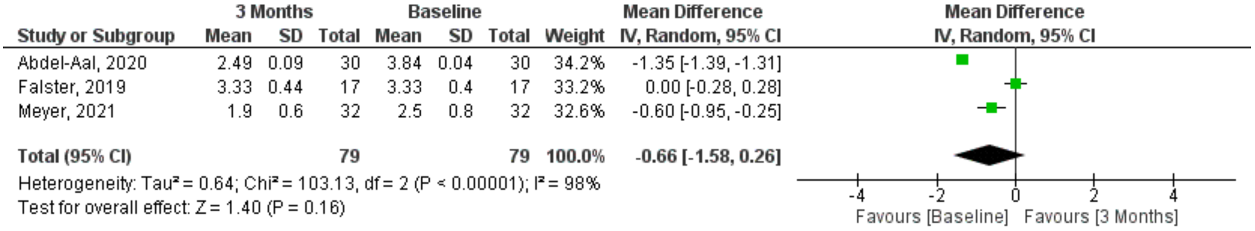

Figure 15S. Meta-analysis results of the proportion of satisfaction.

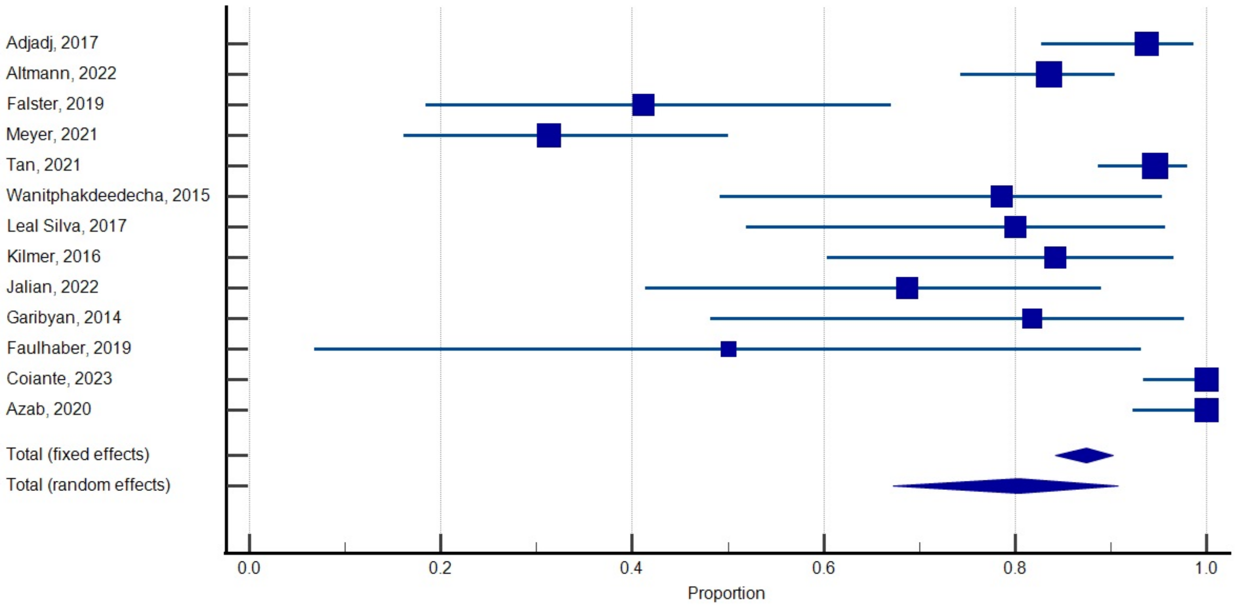

Figure 16S. Meta-analysis results of the proportion of numbness.

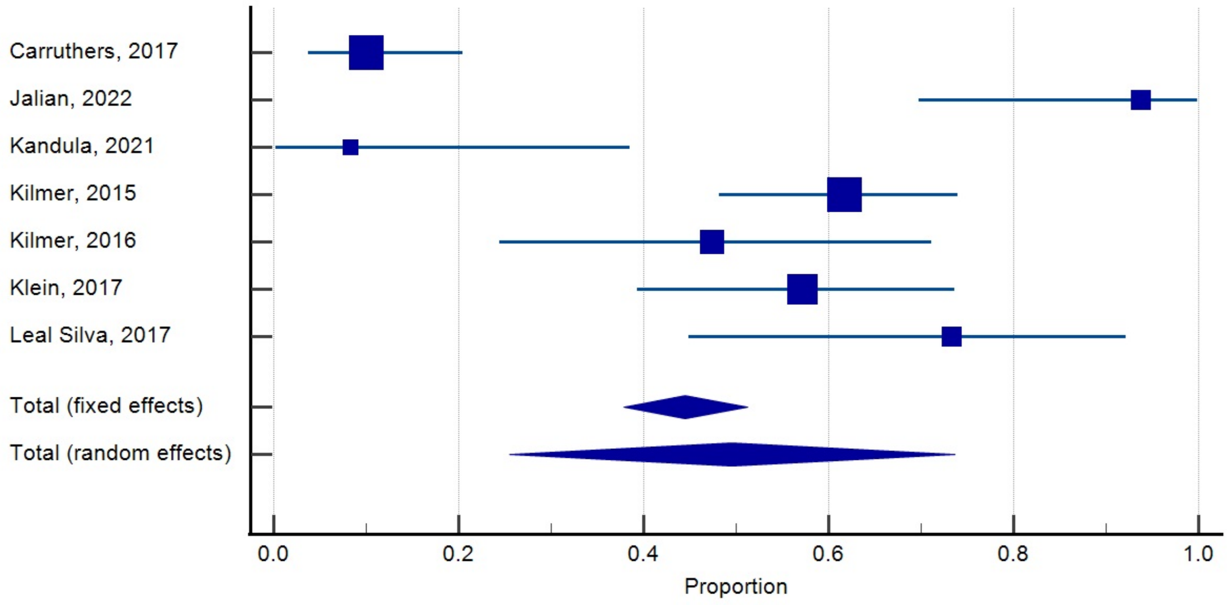

Figure 17S. Meta-analysis results of the proportion of erythema.

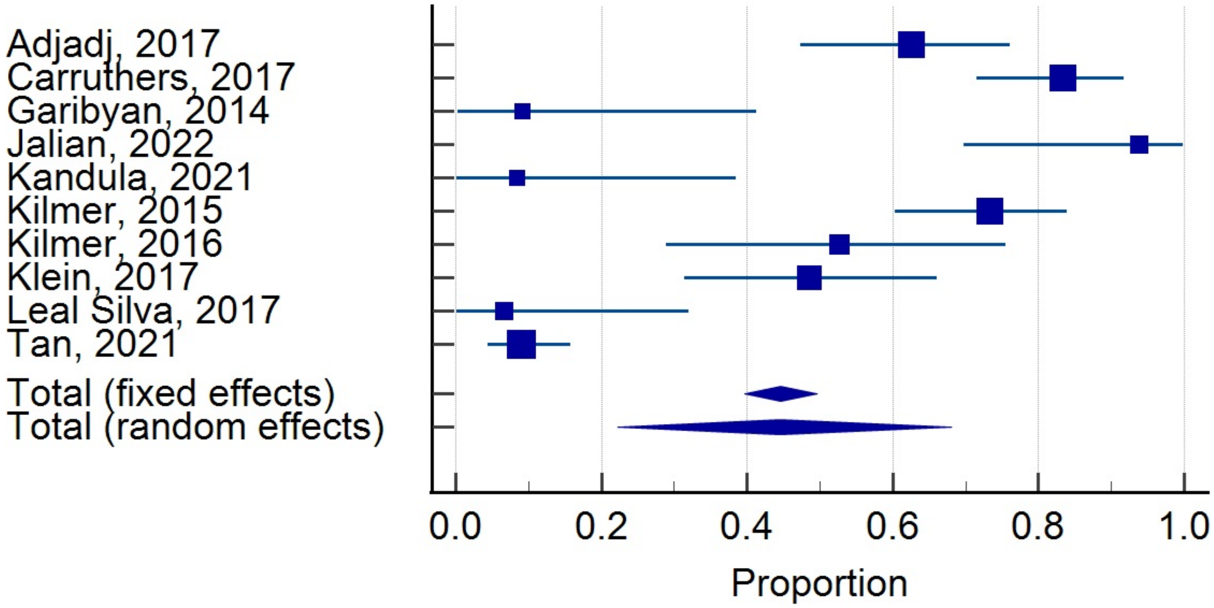

Figure 18S. Meta-analysis results of the proportion of edema.

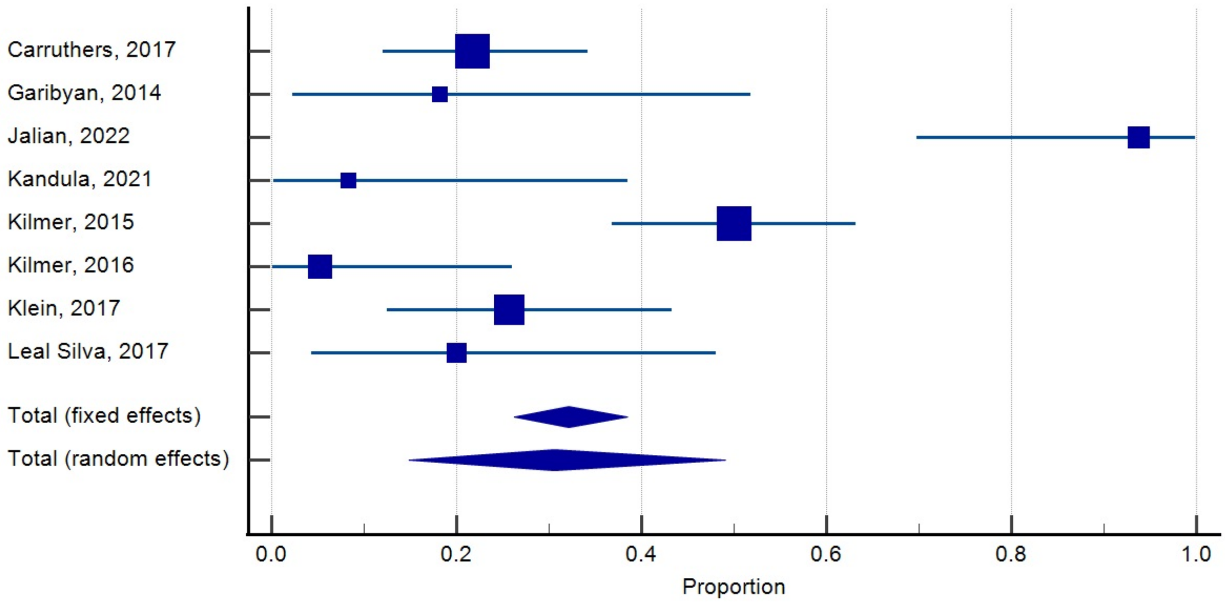

Figure 19S. Meta-analysis results of the proportion of pain.

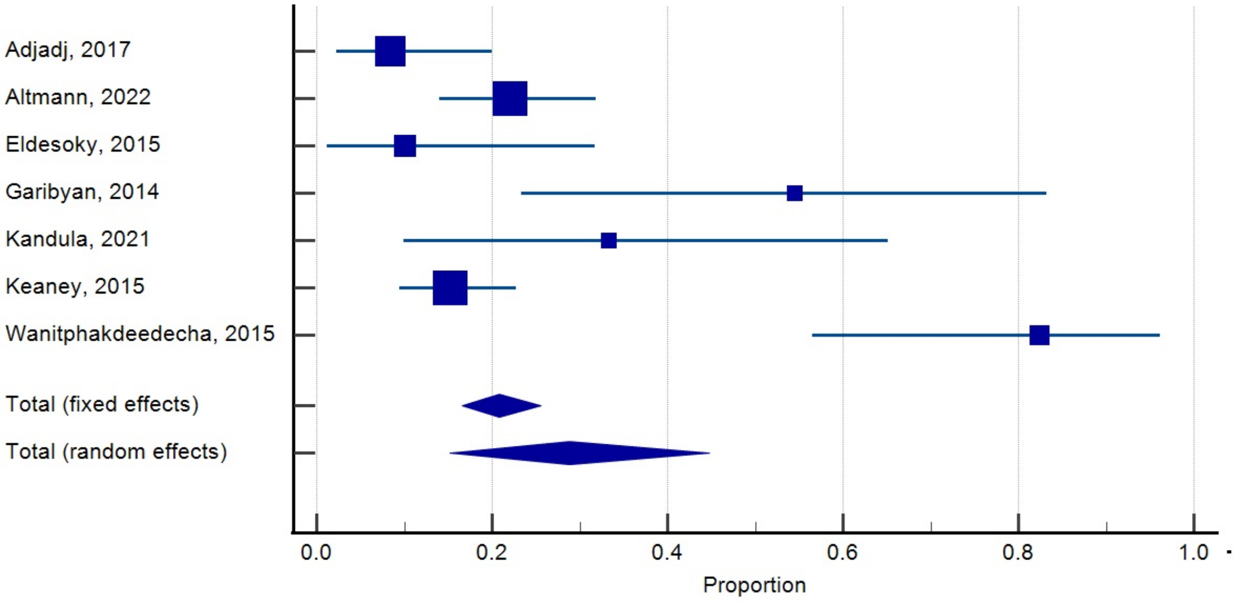

Figure 20S. Meta-analysis results of the proportion of sensitivity.

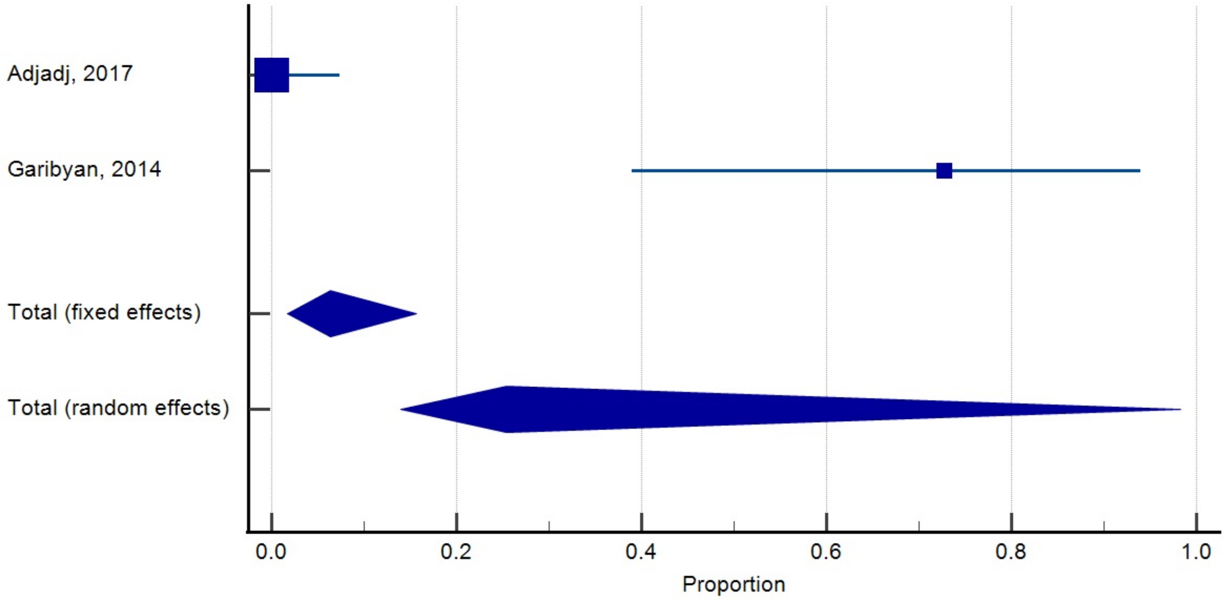

Figure 21S. Meta-analysis results of the proportion of tingling.

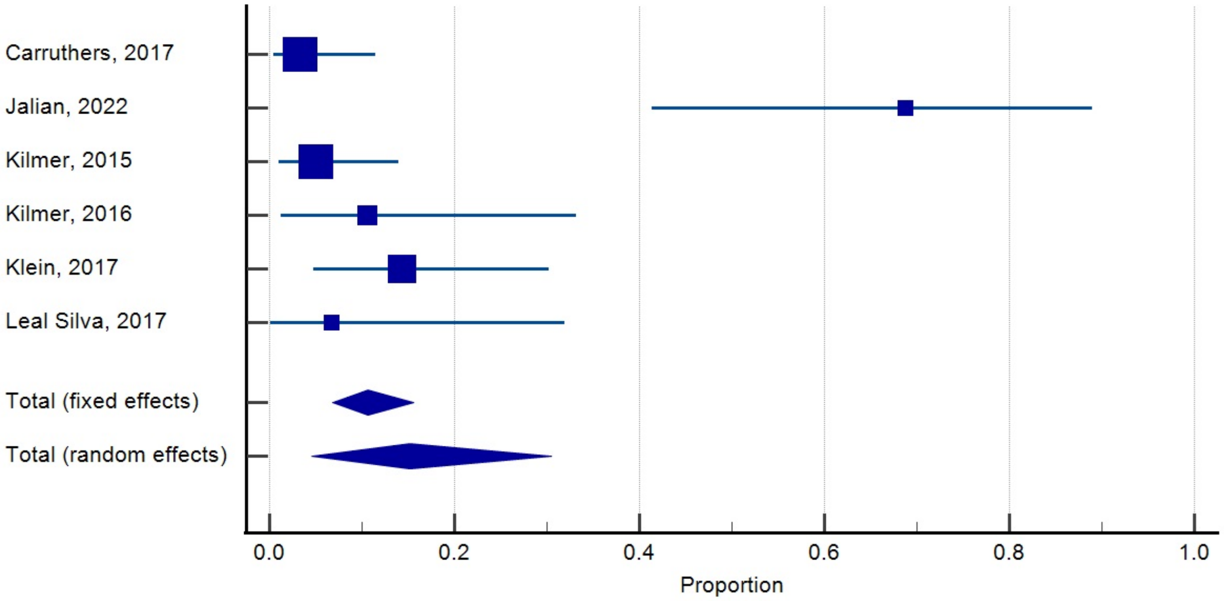

Figure 22S. Meta-analysis results of the proportion of hyperpigmentation.

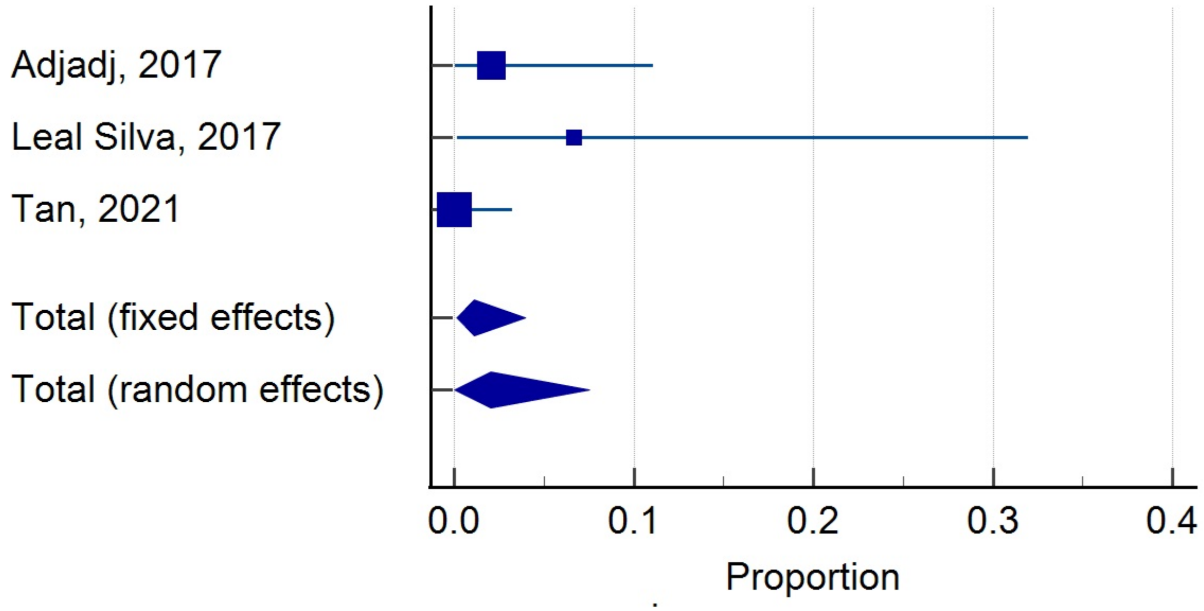

Figure 23S. Funnel plot for publication bias: satisfaction

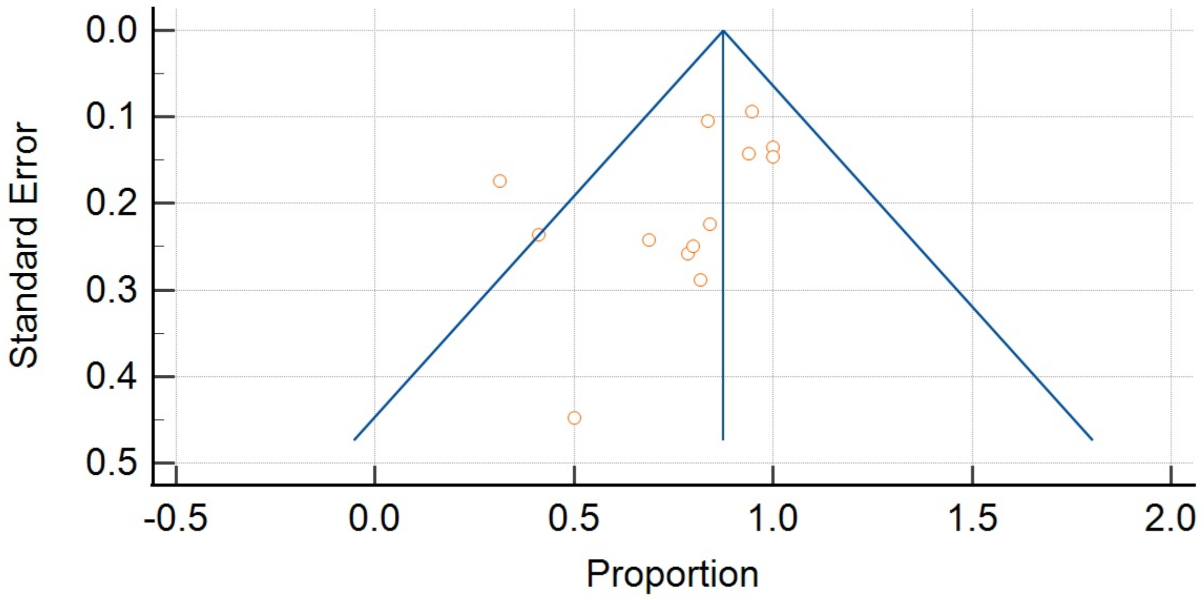

Figure 24S. Funnel plot for publication bias: erythema.

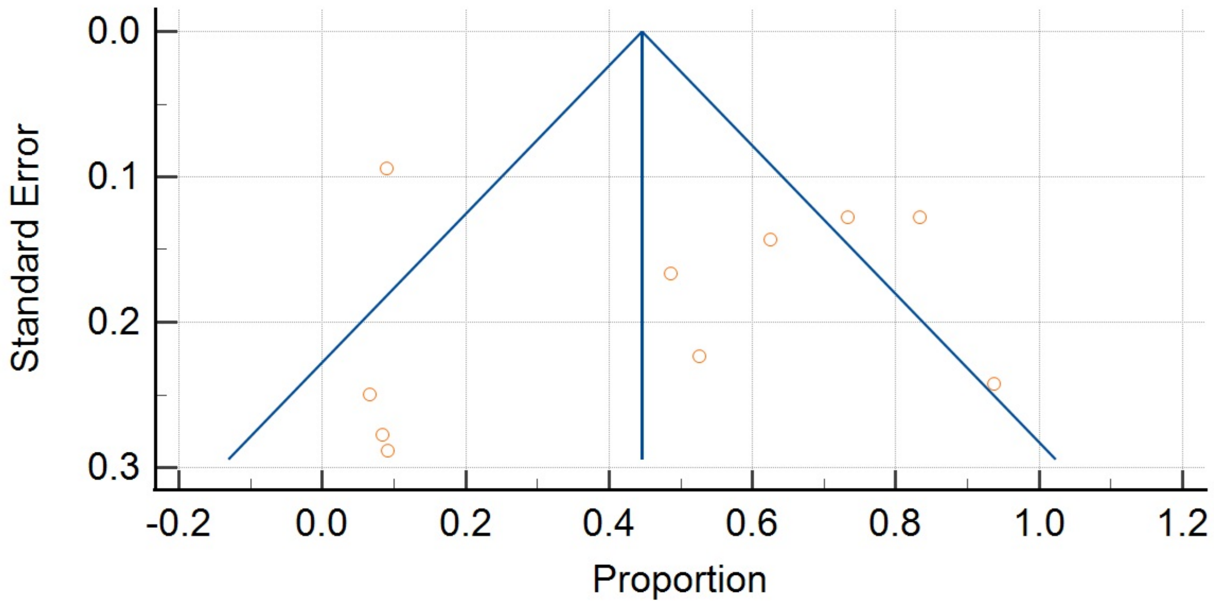

Supplement: Supplementary file 1 — Table S1. PRISMA 2020 Checklist Table S2. Quality assessment and publication bias evaluation of included study using the Newcastle‐Ottawa Scale (NOS) Figure S1. Meta‐analysis of the BMI 12 weeks after cryolipolysis as compared with baseline values. Figure S2. Meta‐analysis of the WHR 12 weeks after cryolipolysis as compared with baseline values. Figure S3. Meta‐analysis of the mean abdominal circumference (cm) 12 weeks after cryolipolysis as compared with baseline values. Figure S4. Meta‐analysis of the mean suprailiac FT (mm) 12 weeks after cryolipolysis as compared with baseline values. Figure S5. Meta‐analysis of the mean thigh circumference (cm) 12 weeks after cryolipolysis as compared with baseline values. Figure S6. Meta‐analysis of the mean subcutaneous FT (mm) 12 weeks after cryolipolysis as compared with baseline values. Figure S7. Meta‐analysis of the mean thigh FT (mm)12 weeks after cryolipolysis as compared with baseline values. Figure S8. Meta‐analysis of the total cholesterol (mg/dl) 12 weeks after cryolipolysis as compared with baseline values. Figure S9. Meta‐analysis of the triglyceride (mg/dl) 12 weeks after cryolipolysis as compared with baseline values. Figure S10. Meta‐analysis of the HDL (mg/dl) 12 weeks after cryolipolysis as compared with baseline values. Figure S11. Meta‐analysis of the LDL (mg/dl) 12 weeks after cryolipolysis as compared with baseline values. Figure S12. Meta‐analysis of the ALT (mg/dl) 12 weeks after cryolipolysis as compared with baseline values. Figure S13. Meta‐analysis of the ALT (mg/dl) 12 weeks after cryolipolysis as compared with baseline values. Figure S14. Meta‐analysis of the abdominal sonography fat thickness (cm) 12 weeks after cryolipolysis as compared with baseline values. Figure S15. Meta‐analysis results of the proportion of satisfaction. Figure S16. Meta‐analysis results of the proportion of numbness. Figure S17. Meta‐analysis results of the proportion of erythema. Figure S18. Meta‐analysis results of the [file OBR-26-e13925-s001.pdf]
